# Phylogenetic Identification, Diversity, and Richness of *Aspergillus* from Homes in Havana, Cuba

**DOI:** 10.3390/microorganisms9010115

**Published:** 2021-01-06

**Authors:** Kenia C. Sánchez Espinosa, Michel Almaguer Chávez, Esperanza Duarte-Escalante, Teresa Irene Rojas Flores, María Guadalupe Frías-De-León, María del Rocío Reyes-Montes

**Affiliations:** 1 Departamento de Microbiología y Virología, Facultad de Biología, Universidad de La Habana, 25, Número 455, Entre I y J, La Habana 10400, Cuba; ksanchez8909@gmail.com (K.C.S.E.); michelalm@fbio.uh.cu (M.A.C.); trojas@fbio.uh.cu (T.I.R.F.); 2 Departamento de Microbiología y Parasitología, Facultad de Medicina, Universidad Nacional Autónoma de México (UNAM), Ciudad Universitaria No. 3000, Ciudad de México 04510, Mexico; dupe@unam.mx; 3 Hospital Regional de Alta Especialidad de Ixtapaluca, Carretera Federal México-Puebla Km. 34.5, Pueblo de Zoquiapan, Ixtapaluca 56530, Mexico; magpefrias@gmail.com

**Keywords:** indoor environments, fungi, *benA*, polyphasic taxonomy

## Abstract

*Aspergillus* is one of the most common fungal genera found indoors; it is important because it can cause a wide range of diseases in humans. *Aspergillus* species identification is based on a combination of morphological, physiological, and molecular methods. However, molecular methodologies have rarely been used for the identification of environmental isolates of *Aspergillus* in Cuba. Therefore, the objective of this work was to identify the species of the genus *Aspergillus* obtained from houses in Havana, Cuba, through the construction of phylogeny from a partial sequence of the *benA* gene region, and to analyze the diversity and richness of *Aspergillus* in the studied municipalities. Isolates of *Aspergillus* spp. included in this study presented the typical macro- and micromorphology described for the genus. According to this polyphasic characterization, *A. niger*, *A. flavus*, *A. welwitschiae*, *A. heteromorphus*, *A. sydowii*, *A. tamarii*, *A. fumigatus*, *A. clavatus,* and *A. tubingensis* were the most abundant species. Most of the identified species constitute new records for outdoor and indoor environments in Cuba and contribute to the knowledge of fungal biodiversity in the country. These results constitute an alert for the health authorities of the country, since prolonged exposure of the inhabitants to *Aspergillus* spores can cause severe persistent asthma, among other diseases.

## 1. Introduction

Indoor environments have unique fungal communities that are adapted to the specific carbon, temperature, and humidity restrictions of these environments [1,2]. The most frequently isolated fungi belong to the *Aspergillus*, *Cladosporium,* and *Penicillium* genera [3,4]. However, *Aspergillus* has particular importance because it can produce a wide range of diseases in humans [5], as the genus *Aspergillus’* members are opportunistic pathogens that pose major threats to immunocompromised individuals, who can acquire the spores of *Aspergillus* spp. present in the environment through inhalation. However, the spores are not effectively eliminated and can remain in the airways, causing a variety of infections, including allergic bronchopulmonary aspergillosis (ABPA), aspergilloma (chronic aspergillosis), and invasive aspergillosis (IA) [6,7].

This genus includes a large number of species that may be responsible for allergic syndromes, intoxications, chronic infections, and acute invasive diseases, particularly in people with compromised immune systems [8,9,10]. The ability to grow and sporulate on various organic substrates allows the widespread development of *Aspergillus* in many geographical areas [11,12,13,14,15]. The carbon sources available for these fungi include damaged building materials, textiles, various food products, and dust [16,17]. At least 428 species have been described in at least 20 groups or sections of *Aspergillus* [18,19,20]. The *Aspergillus* species of the *Circumdati*, *Clavati*, *Cremei*, *Nigri*, *Restricti*, *Usti*, and *Versicolores* sections have been predominantly isolated from the environment, whereas the species of the *Fumigati*, *Terrei*, and *Flavi* sections have been predominantly isolated from clinical samples [8]. The *Aspergillus* species most frequently reported indoors are *A. flavus*, *A. niger*, *A. fumigatus*, *A. calidoustus*, *A. penicillioides*, *A. restrictus*, *A. sydowii*, *A. versicolor*, and *A. westerdijkiae* [1,21,22]. The presence of these species in indoor environments is generally due to the flow of fungal propagules from outside and anthropic activities [23].

In Cuba, the presence of *Aspergillus* has been reported in indoor and outdoor environments, causing allergic diseases in the population [24,25,26]. However, in these studies, the fungus has only been identified based on morphological criteria. Samson et al. [19] defined the criteria for the identification of *Aspergillus* species, which includes the combination of morphological, physiological, molecular, and phylogenetic methods. Within the molecular methods, they recommend using several genetic markers, namely ITS, calmodulin (*CaM*), β-tubulin (*benA*, tub-2), and actin (Act). The International Society for Human and Animal Mycoses has recommended using the ITS region of the *Aspergillus* genus for identification [27]. However, because this region is not sufficiently polymorphic, a secondary identification marker is needed to correctly identify the species. For this, they recommend using *benA* and *CaM*, since they have been shown to be useful in the taxonomy of *Aspergillus* [8].

The objective of this work is to identify the species of the *Aspergillus* genus obtained from homes in Havana, Cuba, through the construction of phylogeny from a partial sequence of the *benA* gene region, and to analyze the diversity and richness of *Aspergillus* in the municipalities studied.

## 2. Materials and Methods 

### 2.1. Fungal Isolates

A total of 153 *Aspergillus* isolates were collected for this biodiversity study. From the indoor environments, 122 isolates were analyzed: 30 from dust samples, 8 from walls, and 84 from the air. For the analysis of the outdoor samples, 31 isolates were collected from homes with a history of moisture in the municipalities of Cerro (23°05′17.0′′ N; 82°23′42.0′′ W) and Arroyo Naranjo (23°02′37′′ N; 82°19′58′′ E), in Havana, Cuba (Table 1).

### 2.2. Sampling Procedure

The air fungal propagules were collected using a SAS Super 100 ™ volumetric biocollector (VWR International Srl, Milano, Italy). The sampler was placed at a height of 1.0 m from the floor, in the center of each sampling area (terrace, patio, or balconies of houses). The medium used was malt extract agar (MEA). Dust was sampled using a sterile brush, whereas the walls were sampled with a swab in places where there was evident fungal growth. Samples were suspended in saline solution and 0.05% Tween 80. An aliquot of each sample’s suspension was cultured on MEA.

### 2.3. Monosporic Culture Procedure

From each isolate grown in potato dextrose agar (PDA) (Bioxon, Ciudad de México, Mexico) for 2–4 days at 28 °C, a conidial suspension was prepared with 1 mL phosphate buffer (pH 7.4) and 0.5% Tween 20 (PBST). This suspension was diluted 1:1000 with PBST, and 50 μL was taken and inoculated in Petri dishes with PDA (Bioxon, Ciudad de México, Mexico). The Petri dishes were then incubated at 28 °C. An isolated colony was selected from each plate and grown in a tube with the same medium at 28 °C. Conidia from the monosporic cultures were stored in sterile distilled water at 4 °C. In addition, all the isolates were stored in tubes with agar at 4 °C or in distilled water and glycerol with skim milk at −30 and −70 °C, respectively, in the Microbial Cultures Collection of the Faculty of Biology, University of Havana, and in the Collection of the Molecular Mycology Laboratory, Mycology Unit, Department of Microbiology and Parasitology, Faculty of Medicine, National Autonomous University of Mexico.

### 2.4. Macro- and Micromorphology

The morphological characterization was performed following the procedures of Klich and Pitt [28] and Samson et al. [19]. The isolates were inoculated in Czapek yeast autolysate (CYA) extract agar and incubated at 5, 25, and 37 °C and in MEA and CYA with 20% sucrose (CYA20S) at 25 °C. The isolates were inoculated at three points on each medium’s plates and incubated at 25 and 37 °C in the dark for 7 days. For micromorphological observations, microscopic mounts were constructed in lactic acid from MEA colonies, and a drop of alcohol was added to remove air bubbles and excess conidia [19].

### 2.5. Obtaining DNA

The isolates were grown in PDA (Bioxón, Ciudad de México, Mexico) for 3 days at 28 °C. From these cultures, a conidial suspension was obtained by adding 1 mL PBS buffer with 0.5% Tween^®^ 20. Subsequently, the 1% yeast extract, 2% peptone, and 2% dextrose (YEPD) was seeded in the liquid medium and incubated at 37 °C with stirring for 2 days or until mycelial growth was observed. The mycelial biomass of each isolate was filtered (with 2 washes performed using sterile milliQ^®^ water, (Merck Millipore, Darmstadt, Germany) under a vacuum with sterile filter paper in a Büchner funnel until the moisture was removed.

DNA was extracted with a Wizard^®^ genomic DNA purification kit (Promega, Madison, WI, USA) as follows: 100 mg of dry mycelial mass from each isolate was deposited in 1.5 mL microtubes containing 0.2 g glass beads, 600 μL lysis solution was added, and the tubes were placed in a FastPrep^®^24 device (MP Biomedicals, Santa Ana, CA, USA). Four periods of cell disruption (40 s at 6 m/s) were applied, with 5 min of cooling between each one after breaking apart the mycelium. We followed the kit’s manufacturer’s instructions. The concentration of the DNA obtained was quantified by spectrophotometry (spectrophotometer DS-11, DeNovix, Wilmington, DE, USA) and gel electrophoresis (1% agarose) and compared against different concentrations (10, 30, and 50 ng/µL) of phage λ (Gibco BRL^®^, San Francisco, CA, USA). Each DNA sample was mixed with 2 µL GelRed ™ nucleic acid gel stain 10,000× (Biotium Inc., Fremont, CA, USA) before being loaded into the gel *benA* PCR.

The following conditions were followed for the PCR: The reaction mixture (25 µL) consisted of 5 ng/µL DNA, 200 mM dNTPs, 1.5 mM MgCl_2_, 30 pmol each of oligonucleotides Bt2a (5′-GGTAACCAAATCGGTGCTGCTTTC-3′) and Bt2b (5′-ACCCTCAGTGTAGTGACCCTTGGC-3′) (Sigma-Aldrich, St. Louis, MO, USA) [29], and 1 U of Taq polymerase (Invitrogen, San Diego, CA, USA) in 1× buffer. The amplification was performed in a thermal cycler (Esco, Swift Maxi, Hatboro, PA, USA) with the following conditions: 95 °C for 8 min, 35 cycles of 95 °C for 15 s, 55 °C for 20 s, and 72 °C for 1 min, and one cycle of 72 °C for 5 min. The amplification products were analyzed by 1.5% agarose gel electrophoresis in 0.5× (45 mM Tris-Base, 45 mM boric acid, 1 mM EDTA (TBE) buffer with a pH of 8.0.

### 2.6. Phylogenetic Analysis

The amplified fragments obtained from the 153 *Aspergillus* isolates were sent to be sequenced in both directions (Macrogen, Rockville, MD, USA). The electropherograms of the obtained sequences were edited using the BioEdit program [30]. The sequences (forward and reverse) for each sample were checked, verified, and used to create consensus sequences, which were subsequently deposited in the GenBank database (http://www.ncbi.nlm.nih.gov) (Supplementary Table S1).

Each edited sequence was compared with all the nucleotide sequences belonging to fungi deposited in GenBank (URL4) through the Basic Local Alignment Search Tool (BLAST) program [31] to evaluate the similarity percentages, identities, and expectation values. The reference sequences identified with the highest similarity and identity percentages, as well as the expectation values closest to zero, were chosen to perform the phylogenetic analysis.

The maximum likelihood method was used to identify the *Aspergillus* species. The support values of the internal branches were assessed using a bootstrap method with 1000 replications (values equal to or higher than 70% were considered significant) and the GTR G+I evolutionary model; the nearest neighbor interchange (NNI) heuristic method was applied. Maximum likelihood (ML) analysis was conducted with MEGA v.10.1.7 software [32].

### 2.7. Richness, Abundance, and Diversity of Species

Species richness (S = the total number of species), abundance (the sum of the number of isolates of each species), and diversity were evaluated through the Shannon diversity index (H’ = –Σ pi Ln pi, where pi is the proportion of individuals of species i) and the Simpson diversity index (D = Ʃ(n/N) 2, where n is the total number of organisms of a particular species, and N is the total number of organisms of all species). The analyses were performed independently for each municipality (Cerro and Arroyo Naranjo) [33,34].

## 3. Results

### 3.1. Phenotypic Identification

All the studied *Aspergillus* isolates presented the typical macro- and micromorphology described for the respective species. Species from the following sections were identified: Section *Versicolores* (13 isolates), section *Usti* (1 isolate), section *Flavi* (33 isolates), section *Aspergillus* (2 isolates), section *Cremei* (1 isolate), section *Fumigati* (10 isolates), section *Clavati* (10 isolates), section *Nigri* (69 isolates), section *Candidi* (1 isolate), section *Flavipedes* (4 isolates), and section *Circumdati* (9 isolates) (Table 2). 

### 3.2. Phylogenetic Analysis

The tree obtained through the maximum likelihood method showed a topology of XXVII groups (Supplementary Figure S1 and Table S1). Group I included 23 isolates of *A. welwitschiae*; group II, 18 isolates of *A. niger*; group III, 2 isolates of *A. neoniger*; group IV, 5 isolates of *A. tubingensis*; group V, 17 isolates of *A. heteromorphus*; group VI, 2 isolates of *A. aculeatinus*; group VII, 1 isolate of *A. tritici*; group VIII, 2 isolates of *A. chevalieri*; group IX, 1 isolate of *A. subramanianii*; group X, 3 isolates of *A. westerdijkiae*; group XI, 1 isolate of *A. ochraceus*; group XII, 4 isolates of *A. melleus*; group XIII, 1 isolate of *A. wentii*; group XIV, 1 isolate of *A. calidoustus*; group XV, 2 isolates of *A. versicolor*; group XVI, 11 isolates of *A. sydowii*; group XVII, 1 isolate of *A. templicola*; group XVIII, 3 isolates corresponding to *A. micronesiensis*; group XIX, 11 isolates corresponding to *A. tamarii*; group XX, 1 isolate of *A. nomius*; group XXI, 1 isolate of *A. oryzae*; group XXII, 20 isolates of *A. flavus*; group XXIII, 10 isolates of *A. fumigatus*; group XXIV, 2 isolates of *A. giganteus*; group XXV, 8 isolates of *A. clavatus*; group XXVI, 1 isolate of *A. aculeatus*; and group XXVII, 1 isolate of *A. violaceofuscus.*

### 3.3. Richness, Abundance, and Diversity of Species

**Figure 1:**
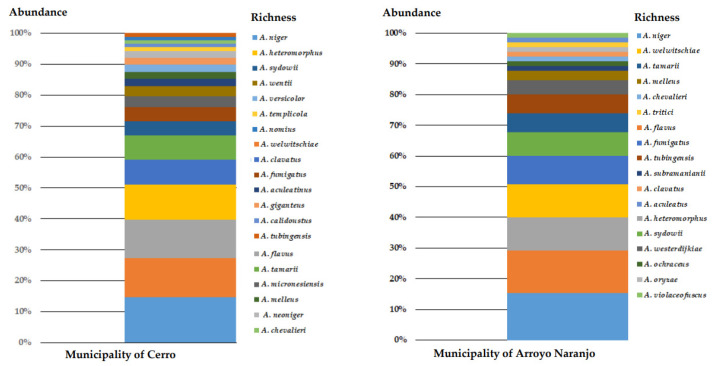
Richness and abundance (%) of *Aspergillus* species in the sampled areas of the Cerro and Arroyo Naranjo municipalities.

The results showed higher species richness in the municipality of Cerro, from which 20 species were identified, whereas in the municipality of Arroyo Naranjo, 18 species were identified. Likewise, the results showed that in the municipality of Cerro, *A. niger* (14.77%), *A. welwitschiae* (12.5%), *A. flavus* (12.5%), *A. heteromorphus* (11.36%), *A. clavatus* (7.96%), and *A. tamarii* (7.96%) were the most abundant species (Figure 1), whereas *A. niger* (15.38%), *A. flavus* (13.85%), *A. heteromorphus* (10.77%), *A. welwitschiae* (10.77%), *A. fumigatus* (9.23%), and *A. sydowii* (7.69%) were the most abundant species in the Arroyo Naranjo municipality (Figure 1).

The diversity, estimated through the Shannon and Simpson indices, was slightly greater in the municipality of Cerro than in Arroyo Naranjo (Table 3).

## 4. Discussion

A high prevalence of allergic diseases has been observed in Cuba. de la Vega Pazitková et al. [35] showed that 9% of young people under the age of 15 in Havana might suffer from undiagnosed asthma. Venero Fernández et al. [36] estimated that allergic diseases have a prevalence of 32%, although the triggering factors were not well-established in both studies. However, this high prevalence can be explained by the climatic conditions in Havana, as it has an average annual temperature of 25.5 °C, average annual precipitation of 1510 mm, and a relative humidity of 78%. These weather conditions are shared by other tropical countries, which contribute to fungal development, associated with an increased number of spores in the environment [37].

An indoor and outdoor air myco-biological study performed in housing environments located in Havana, Cuba, showed that *A. flavus*, *A. niger,* and *A. clavatus* were the most frequently isolated species [37]. In addition, other studies have focused on fungi identification in indoor or outdoor environments in Havana [38,39]; however, none used genotypic methods for identification at the species level. Therefore, identifying *Aspergillus* isolates at the species level through molecular methods represents a significant contribution, since these may be associated with allergic conditions.

Here, we identified *Aspergillus* isolates obtained from indoor and outdoor housing environments in Havana, Cuba, belonging to the following sections: *Versicolores*, *Usti*, *Flavi*, *Aspergillus*, *Cremei*, *Fumigati*, *Clavati*, *Nigri*, *Candidi*, *Flavipedes,* and *Circumdati*. Of these, species from the sections *Fumigati*, *Flavi*, and *Nigri* may cause allergic diseases [40]; they were also found to be the most abundant.

The species identified in the indoor housing environments coincided with those detected in the outdoor environments, which is consistent with the results described by Rahmawati et al. [2]. The movement of spores can explain this coincidence from outdoor environments; spores enter houses through open windows and doors, on the clothing of inhabitants and visitors, or through the entry and exit of pets [5]. Initially, in this work, the isolates were identified according to their macro- and microscopic characteristics. However, these methods have limitations due to the morphological similarity and variability shown by these fungi; therefore, it was only possible to identify the *Aspergillus* isolates at the section level. Thus, genotypic methods were needed to correctly identify the *Aspergillus* species, and the *benA* marker was used to construct a phylogenetic analysis through a maximum likelihood method.

According to this polyphasic identification, *A. welwitschiae* (section *Nigri*), *A. flavus* (section *Flavi*), *A. niger* (section *Nigri*), *A. heteromorphus* (section *Nigri*), *A. sydowii* (section *Versicolores*), *A. tamarii* (section *Flavi*), *A. fumigatus* (section *Fumigati*), *A. clavatus* (section *Clavati*), and *A. tubingensis* (section *Nigri*) were the most abundant species. Section *Nigri* was the most represented among the isolates evaluated. These results are different from those reported by Richardson et al. [41], who most frequently isolated *A. fumigatus* among the species of this genus. Hashimoto et al. [42], when identifying clinical and environmental isolates of the *Nigri* section, reported that this set of isolates included three species, *A. niger*, *A. welwitschiae*, and *A. tubingensis*, of which *A. welwitschiae* had the highest number of isolates identified, which is consistent with our results. This may correspond to the members of these sections being xerotolerant and thermotolerant, so they grow in a wide range of temperatures [43]. In other studies, *A. niger* was identified and showed a frequency similar to that obtained in this study, but in hospital settings [44]. This supports the importance of the presence of species from the section *Nigri* in the environment, as that section includes species that cause pulmonary aspergillosis and otomycosis in humans, as well as localized and disseminated diseases in domestic and wild animals [45].

*Aspergillus niger*, *A. welwitschiae*, and *A. tubingensis* are distributed worldwide, whereas the rest of the species identified in this work have only been reported in contaminated food, in the soil, and in the air in some countries [22,46]. *A. heteromorphus, A. neoniger,* and *A. aculeatinus* have been described as new global records in the air of indoor and outdoor environments, and *A. heteromorphus* and *A. violaceofuscus* in dust [1]. *A. aculeatus* and *A. violaceofuscus* have only been previously detected in the indoor air of libraries in Brazil [22].

The detection of the species in the section *Nigri* is important since, as several authors have stated, *A. welwitschiae* and *A. niger* have the ability to produce ochratoxin A (OTA), a potent nephrotoxin that causes carcinogenic effects and fumonisin [47]. Although few of the species in the section *Nigri* are involved in mycoses associated with humans or animals, there are reports of IA caused by *A. niger* [46], onychomycosis caused by *A. welwitschiae*, *A. tubingensis*, and *A. niger* [48] and external otitis caused by *A. niger* and *A. tubingensis* [8].

The species *A. flavus*, *A. tamarii, A. oryzae*, and *A. nomius* identified in this study belong to the section *Flavi*; they are considered cosmopolitan, although *A. nomius* has only been previously recorded in indoor environments in Brazil [1,22]. *A. nomius* has been reported to produce several aflatoxins and have a carcinogenic effect [49]. *A. flavus* produces aflatoxins B1 and G, aflatrem, cyclopiazonic acid, and 3-nitropropionic acid; *A. tamarii* produces tenuazonic acid, cyclopiazonic acid, and 3-nitropropionic acid; whereas *A. nomius* produces tenuazonic acid, and aflatoxins B1 and G [50]. However, reports of aspergillosis due to *A. tamarii* and *A. nomius* are scarce, since their conidia tend to be deposited in the sinuses and in the upper respiratory areas [51]. *Aspergillus oryzae* is the domesticated form of the aflatoxigenic species *A. flavus* [50]. *A. flavus* is considered the etiologic agent responsible for 10% of bronchopulmonary aspergillosis worldwide and the second most important agent of IA. The high concentrations of its conidia in the outdoor and indoor environments of houses and hospitals have been correlated with the diseases it causes in tropical countries [15].

*Aspergillus ochraceus, A. westerdijkiae*, and *A. subramanianii*, belonging to the section *Circumdati*, represent the first records of these species in environmental studies in the Caribbean. *A. melleus* was detected by Rojas et al. [21] in the library of the Faculty of Biology in Havana. *A. ochraceus* and *A. westerdijkiae* are of clinical importance, since they have been reported as causing chronic granulomatous diseases and non-dermatophyte onychomycosis, respectively [52,53]. *Aspergillus subramanianii* has been reported as a causal agent of IA, exclusively for patients with inborn defects in the host antifungal defense pathways [53].

The species *A. sydowii* and *A. versicolor* (section *Versicolores*) are commonly isolated from indoor environments, and *A. versicolor* has been associated with sick building syndrome [1]. These species have also been recognized as emerging pathogens in onychomycosis [54,55]. In addition, *A. versicolor* can cause invasive pulmonary aspergillosis and produce OTA [15].

The presence of the species *A. clavatus* and *A. giganteus* (section *Clavati*) is common in tropical, subtropical, and Mediterranean regions, whereas *A. giganteus* has not been reported in tropical or subtropical regions. Several studies have reported that *A. clavatus* is the cause of endocarditis, extrinsic allergic alveolitis, and toxic syndromes with neurological disorders [56].

Notably, records in the Caribbean of the species *A. micronesiensis* and *A. templicola* are limited. These species included in the *Flavipedes* section were first described by Visagie et al. [1] in the dust of homes in Mexico and Thailand. Likewise, Visagie et al. [1] reported *A. micronesiensis* in the dust of North American homes. However, in our study, except for the CCMFBH-917 isolate from an indoor environment, the majority of the isolates were obtained from dust.

The species *A. calidoustus* (section *Usti*) constitutes a new record for Latin America, since it has only been reported from indoor environments in the United States, West Asia, and Europe, and in the dust of North American homes [1,57]. *A. calidoustus*, usually confused with *A. ustus*, is an emerging pathogen that causes IA [8,58].

Two isolates identified as *A. chevalieri* (section *Aspergillus*) were obtained from indoor environments. Júnior et al. [22] showed that their presence is common in these environments. Recently, Siqueira et al. [59] reported this species as a causal agent of fatal cutaneous and cerebral aspergillosis following traumatic inoculation.

The species *A. fumigatus* (section *Fumigati*) was the only species identified in the *Fumigati* section. This fungus produces large numbers of conidia (asexual spores), which easily become airborne and are efficiently dispersed through the air due to their small size (2–3 μm diameter) and inherent hydrophobicity. Additionally, these conidia persist in the air for long periods due to the complex nature of the cell wall, which protects them from several physical and chemical stressors [60]. This species, due to its multiple pathogenic attributes, can cause IA, aspergilloma, and ABPA in immunocompromised individuals [61]. In addition, prolonged exposure to *A. fumigatus* conidia in indoor housing environments can cause occupational asthma [10].

The sections *Cremei* and *Candidi* were each represented by a single species: *A. wentii* and *A. tritici*, respectively. *A. wentii* was previously reported in the indoor environment of the Central Library of Havana University [21]. *A. tritici* was not found on the walls of houses in any geographical region. There are few reports of *A. tritici* associated with medical pathologies, possibly due to its morphological similarity to *A. candidus*. Hubka et al. [62] reported the first case of *A. tritici* associated with onychomycosis. They suggested that it could have been the same agent previously associated with onychomycosis, and that it was possibly misidentified as *A. candidus* in other reports due to a lack of sequencing analysis.

The *benA* marker has been previously used to study the phylogeny within the *Aspergillus* genus and other related species [16] because it is a conserved, slow-evolving gene with a high degree of interspecific variability in the intronic regions. In the present study, the *benA* marker was used to classify the environmental isolates of *Aspergillus* spp., corroborating its value as a phylogenetic marker for species identification. In another study, Duarte-Escalante et al. [63] carried out the phenotypic identification of isolates of *A. fumigatus* from Mexico, Argentina, France, and Peru, assigning all isolates to the *Fumigati* section, based on their macro- and micromorphology. The phenotypic classification was confirmed by the phylogenetic analysis with sequences of the *benA* gene region, which showed that all isolates belonged to the species *A. fumigatus*. Likewise, when Montenegro et al. [64] characterized the phenotyping and genotyping of isolates of the *Fumigati* section, they used several conserved genes, including the gene that encodes for the b-tubulin-encoding gene (*benA*), the mitochondrial cytochrome b-encoding gene (*mtcytb*), the gene that encodes for rodlet (*rodA*), the salt-responsive gene, the internal transcribed spacer (ITS) 1-5.8S-ITS2 region, the genes encoding calmodulin (*CaM*), and actin. They used partial sequences of these genes to assess the phylogenetic relationships among the *Aspergillus* species; they found that the most relevant genes for differentiating the *Aspergillus* species are *benA* and *rodA.*

Finally, the municipality of Cerro is slightly richer than the municipality of Arroyo Naranjo in the number of *Aspergillus* species and diversity; this small difference can be explained by the two regions having different microenvironmental conditions. In addition, the Cerro municipality is characterized as one of the most industrialized and with the highest vehicular traffic in the province of Havana and is therefore one of the most complex from the environmental point of view.

## 5. Conclusions

Fifteen species identified in this study constitute new records for outdoor and indoor environments in Cuba (*A. calidoustus*, *A. heteromorphus*, *A. nomius*, *A. giganteus*, *A. violaceofuscus*, *A. aculeatus*, *A. neoniger*, *A. welwitschiae*, *A. tritici*, *A. aculeatinus*, *A. micronesiensis*, *A. templicola*, *A. subramanianii*, *A. melleus*, *and A. ochraceus*). This is relevant, since our findings contribute to the knowledge of fungal biodiversity and ecology. In addition, it constitutes an alert for the sanitary authorities of Cuba, since prolonged exposure of the inhabitants to the spores and humidity in houses can aggravate symptoms of asthma and allergic rhinitis in susceptible individuals.

## Figures and Tables

**Table 1:** Origin of the *Aspergillus* isolates from two municipalities of Havana, Cuba.

Municipality	Origin	Isolate
Cerro	Outdoor air	CCMFBH-835, CCMFBH-836, CCMFBH-837, CCMFBH-838, CCMFBH-841, CCMFBH-842, CCMFBH-843, CCMFBH-844, CCMFBH-845, CCMFBH-846, CCMFBH-847, CCMFBH-852, CCMFBH-853, CCMFBH-854, CCMFBH-855, CCMFBH-856, CCMFBH-857, CCMFBH-858, CCMFBH-860, CCMFBH-863, CCMFBH-864, CCMFBH-868, CCMFBH-869
Arroyo Naranjo	Outdoor air	CCMFBH-878, CCMFBH-879, CCMFBH-886, CCMFBH-887, CCMFBH-891, CCMFBH-892, CCMFBH-898, CCMFBH-899
Cerro	Indoor air	CCMFBH-839, CCMFBH-848, CCMFBH-849, CCMFBH-850, CCMFBH-859, CCMFBH-861, CCMFBH-862, CCMFBH-866, CCMFBH-867, CCMFBH-870, CCMFBH-871, CCMFBH-872, CCMFBH-873, CCMFBH-874, CCMFBH-899, CCMFBH-900, CCMFBH-901, CCMFBH-902, CCMFBH-903, CCMFBH-904, CCMFBH-905, CCMFBH-906, CCMFBH-907, CCMFBH-908, CCMFBH-909, CCMFBH-911, CCMFBH-912, CCMFBH-913, CCMFBH-914, CCMFBH-919, CCMFBH-920, CCMFBH-921, CCMFBH-922, CCMFBH-923, CCMFBH-924 CCMFBH-925, CCMFBH-926, CCMFBH-940, CCMFBH-941, CCMFBH-942, CCMFBH-944, CCMFBH-945, CCMFBH-946, CCMFBH-947, CCMFBH-948, CCMFBH-949, CCMFBH-950, CCMFBH-951, CCMFBH-952
Arroyo Naranjo	Indoor air	CCMFBH-875, CCMFBH-876, CCMFBH-880, CCMFBH-881, CCMFBH-883, CCMFBH-884, CCMFBH-885, CCMFBH-888, CCMFBH-889, CCMFBH-890, CCMFBH-893, CCMFBH-895, CCMFBH-896, CCMFBH-916, CCMFBH-917, CCMFBH-918, CCMFBH-927, CCMFBH-928, CCMFBH-929, CCMFBH-930, CCMFBH-932, CCMFBH-933, CCMFBH-934, CCMFBH-935, CCMFBH-936, CCMFBH-937, CCMFBH-938, CCMFBH-939, CCMFBH-953, CCMFBH-954, CCMFBH-955, CCMFBH-996, CCMFBH-997, CCMFBH-1000, CCMFBH-1001
Cerro	Dust	CCMFBH-965, CCMFBH-966, CCMFBH-967, CCMFBH-968, CCMFBH-969, CCMFBH-970, CCMFBH-971, CCMFBH-972, CCMFBH-973, CCMFBH-974, CCMFBH-975, CCMFBH-976, CCMFBH-977, CCMFBH-978
Arroyo Naranjo	Dust	CCMFBH-979, CCMFBH-980, CCMFBH-981, CCMFBH-982, CCMFBH-983, CCMFBH-984, CCMFBH-985, CCMFBH-986, CCMFBH-987, CCMFBH-988, CCMFBH-989, CCMFBH-990, CCMFBH-992, CCMFBH-993, CCMFBH-994, CCMFBH-995
Cerro	Walls	CCMFBH-956, CCMFBH-958
Arroyo Naranjo	Walls	CCMFBH-959, CCMFBH-960, CCMFBH-961, CCMFBH-962, CCMFBH-963, CCMFBH-964

**Table 2:** Distinctive macro and micromorphological features of the studied *Aspergillus* isolates.

Section	Macro- and Micromorphological Characteristics
*Versicolores*	Colony on CYA, 25 °C, 7 days: Variable coloration, greenish, turquoise, yellowish, and radially sulcate; velutinous texture; tawny olive to orange cinnamon reverse, vinaceous or brown or scarlet; moderate to abundant exudate, clear to yellowish to reddish-brown, reddish-brown soluble pigment. Biseriate conidial heads, radiate, globose to subglobose conidia, rough to prickly.
*Usti*	Colony on CYA, 25 °C, 7 days: Blond/greyish-yellow; floccose texture; yellow reverse with a beige center. Biseriate, predominantly pyriform vesicles, globose conidia, very rough ornamentation, with an inner and outer visible wall.
*Flavi*	Colony on CYA, 25 °C, 7 days: Green olive, brown olive, greenish-brown, brownish yellow; woolly or floccose texture; colorless, light brown or orange reverse. Presence of brown sclerotia in some isolates. Biseriate and uniseriate conidial heads, radiated to columnar; rough, colorless stipe, globose to ellipsoidal conidia, rough in some isolates.
*Aspergillus*	Colony on CYA, 25 °C, 7 days: Sulfur yellow mycelium; floccose texture; luteous to ochreous reverse. Uniseriate conidial heads, Ascomata Eurotium-like, cleistothecial, superficial, yellow, globose to subglobose. Ascospores hyaline, globose to subglobose in surface view.
*Cremei*	Colony on CYA, 25 °C, 7 days: Greyish-yellow, smooth texture; brownish-cream reverse. Biseriate, radiated conidial heads separated into columns, smooth and colorless wall stipe, globose or subglobose conidia, smooth to rough.
*Fumigati*	Colony on CYA, 25 °C, 7 days: Greyish-turquoise; velutinous texture; creamy reverse. Uniseriate, predominantly pyriform vesicles, grey to blue-green conidia.
*Clavati*	Colony on CYA, 25 °C, 7 days: Blue-green or pale blue-green; velvety texture; uncolored or dull tan reverse. Presence of exudates in some isolates but no soluble pigments. Uniseriate conidiophores with blue-green conidia, hyaline conidiophore stipes, and clavate aspergilla.
*Nigri*	Colony on CYA, 25 °C, 7 days: Brown to black; floccose texture, sulcate; beige to cream-yellow, brown, dark grey, uncolored to yellow reverse. Uniseriate or biseriate, spherical vesicles, smooth stipes, rough dark globose, finely rough, or finely spiny conidia.
*Candidi*	Colony on CYA, 25 °C, 7 days: Light cream; submerged texture; light brown reverse. Biseriate conidial heads, radiate, thick-walled conidiophores, septate, elongated vesicles, small conidia globose to subglobose, slightly roughened.
*Flavipedes*	Colony on CYA, 25 °C, 7 days: Yellowish white; floccose surface texture; brown to dark brown reverse. Biseriate conidiophores, radiate, elongated or subglobose vesicles, globose to subglobose conidia, smooth to finely roughened.
*Circumdati*	Colony on CYA, 25 °C, 7 days: Yellow to ochre colonies, cream, or pale yellow; smooth texture; yellow, orange, or cream reverse. Presence of pink to purple-brown sclerotia in some isolates. Predominantly biseriate, rough stems, smooth or finely rough globose conidia.

**Table 3:** Fungal diversity in the municipalities of Cerro and Arroyo Naranjo.

Municipality	Simpson Index	Shannon Index
Cerro	0.0864	−1.6688
Arroyo Naranjo	0.0930	−1.1638

## Data Availability

The datasets supporting the conclusion of this article are included within the article and supplementary material files. Nucleotide sequences reported in this article are available via GenBank.

## Supplementary Materials

The following are available online at https://www.mdpi.com/2076-2607/9/1/115/s1, Figure S1: Molecular phylogenetic analysis by the maximum likelihood method. The evolutionary history was inferred using the maximum likelihood method based on the general time-reversible model. The bootstrap consensus tree inferred from 1000 replicates was taken to represent the evolutionary history of the taxa analyzed; Table S1: Groups identified in the phylogenetic tree obtained by maximum likelihood from *benA* gene sequences.
