# Supplementary material for: Phylogenetic Identification, Diversity, and Richness of Aspergillus from Homes in Havana, Cuba
Source: Microorganisms. 2021 Jan 6;9(1):115. doi: 10.3390/microorganisms9010115 (PMC7825327; doi:10.3390/microorganisms9010115)
Supplement: Supplementary file 1 [file microorganisms-09-00115-s001.zip › microorganisms-1059983-sl-revised/Supplementary Table S1 Sanchez Espinosa et al..pdf]

Suplementary Table S1. Groups identified in the phylogenetic tree obtained by maximum likelihood from *BenA* gene sequences

Group	Isolate	Genbank Access Number	Specie	Section
**I**	*MH614486.1 *A. welwitschiae*<br>*MH614483.1-*A. welwitschiae*<br>*MH614497.1 *A. welwitschiae*<br>CCMFBH-845<br>CCMFBH-848<br>CCMFBH-849<br>CCMFBH-852<br>CCMFBH-855<br>CCMFBH-883<br>CCMFBH-901<br>CCMFBH-903<br>CCMFBH-926<br>CCMFBH-928<br>CCMFBH-932<br>CCMFBH-934<br>CCMFBH-936<br>CCMFBH-939<br>CCMFBH-942<br>CCMFBH-944<br>CCMFBH-949<br>CCMFBH-975<br>CCMFBH-981<br>CCMFBH-983<br>CCMFBH-988<br>CCMFBH-994<br>CCMFBH-997	 <br><br>MT410098<br>MT410099<br>MT410100<br>MT410101<br>MT410102<br>MT410103<br>MT410104<br>MT410106<br>MT410105<br>MT410107<br>MT410070<br>MT410108<br>MT410109<br>MT410072<br>MT410110<br>MT410111<br>MT410112<br>MT410113<br>MT410114<br>MT410115<br>MT410116<br>MT410081<br>MT410117	*A. welwitschiae*	***N**igri*
**II**	*MN567299.1 A. *niger*<br>*MH204824.1 A. *niger*<br>*MH208759.1 *A. niger*<br>*MK854747.1 *A. niger*<br>*MG991342.1 A. *niger*<br>CCMFBH-886<br>CCMFBH-887<br>CCMFBH-904<br>CCMFBH-905<br>CCMFBH-906<br>CCMFBH-907<br>CCMFBH-908<br>CCMFBH-922<br>CCMFBH-927	 <br><br><br><br>MT410061<br>MT410062<br>MT410063<br>MT410064<br>MT410065<br>MT410066<br>MT410067<br>MT410068<br>MT410069	*A. niger*	*Nigri*

	CCMFBH-937<br>CCMFBH-946<br>CCMFBH-947<br>CCMFBH-951<br>CCMFBH-952<br>CCMFBH-954<br>CCMFBH-966<br>CCMFBH-971<br>CCMFBH-982	MT410071<br>MT410073<br>MT410074<br>MT410075<br>MT410076<br>MT410078<br>MT410079<br>MT410080		
**III**	*MH614464.1 *A. neoniger*<br>CCMFBH-839<br>CCMFBH-857	 MT410059<br>MT410060	*A. neoniger*	***N**igri*
**IV**	*KT326813.1 *A. tubingensis*<br>*JX545086.1 *A. tubingensis*<br>*KX231820.1 *A. tubingensis*<br>CCMFBH-864<br>CCMFBH-880<br>CCMFBH-955<br>CCMFBH-979<br>CCMFBH-996	 <br><br>MT410082<br>MT410083<br>MT410084<br>MT410085<br>MT410086	*A. tubingensis*	***N**igri*
**V**	*MH614443.1-*A. heteromorphus*<br>*MH614573.1 *A. heteromorphus*<br>*HQ632678.1 -*A. heteromorphus*<br>* AY585529.1-*A. heteromorphus*<br>CCMFBH-836<br>CCMFBH-846<br>CCMFBH-861<br>CCMFBH-867<br>CCMFBH-868<br>CCMFBH-869<br>CCMFBH-871<br>CCMFBH-872<br>CCMFBH-875<br>CCMFBH-881<br>CCMFBH-884<br>CCMFBH-885<br>CCMFBH-900<br>CCMFBH-914<br>CCMFBH-980<br>CCMFBH-998<br>CCMFBH-999	 <br><br><br>MT410118<br>MY410125<br>MT410131<br>MT410134<br>MT410135<br>MT410136<br>MT410137<br>MT410138<br>MT410140<br>MT410142<br>MT410143<br>MT410144<br>MT410149<br>MT410155<br>MT410175<br>MT410180<br>MT410181	*A. heteromorphus*	*Nigri*
**VI**	*EU159220.1-*A. aculeatinus*<br>CCMFBH-909	 MT410151	*A. aculeatinus*	*Nigri*

	CCMFBH-913	MT410154		
**VII**	*EU076293.1-*A. tritici*<br>CCMFBH-959	 MT410168	*A. tritici*	*Candidi*
**VIII**	LT627280.1 *A. chevalieri*<br>LT627272.1 *A. chevalieri*<br>CCMFBH-912<br>CCMFBH-935	 <br>MT410153<br>MT410163	*A. chevalieri*	*Aspergillus*
**IX**	*KJ775050.1-*A. subramanianii*<br>*EF661339.1-*A. subramanianii*<br>*EF661338.1-*A. subramanianii*<br>CCMFBH-890	 <br><br>MT410146	*A. subramanianii*	*Circumdati*
**X**	*FR839686.2-*A. westerdijkiae*<br>*JX535306.1-*A. westerdijkiae*<br>CCMFBH-879<br>CCMFBH-964<br>CCMFBH-995	 <br>MT410141<br>MT410169<br>MT410179	*A. westerdijkiae*	*Circumdati*
**XI**	*EU021662.1-*A. ochraceus*<br>*EF661322.1-*A. ochraceus*<br>CCMFBH-917	 <br>MT410156	*A. ochraceus*	*Circumdati*
**XII**	*FM995525.1 *A. melleus*<br>*FM995526.1 *A. melleus*<br>CCMFBH-838<br>CCMFBH-919<br>CCMFBH-930<br>CCMFBH-989	 <br>MT410119<br>MT410157<br>MT410161<br>MT410177	*A. melleus*	*Circumdati*
**XIII**	*EF652106.1-*A. wentii*<br>CCMFBH-873	 MT410139	*A. wentii*	*Cremei*
**XIV**	*KJ777803.1-*A. calidoustus*<br>CCMFBH-940	 MT410164	*A. calidoustus*	*Usti*
**XV**	* KX455751.1-*A. versicolor*<br>CCMFBH-950<br>CCMFBH-972	 MT410167<br>MT410173	*A. versicolor*	*Versicolores*
**XVI**	* MG831183.1 -*A. sydowii*<br>* KU737555.1 *A. sydowii*<br>CCMFBH-835<br>CCMFBH-862<br>CCMFBH-874	 <br>MT410087<br>MT410088<br>MT410089	*A. sydowii*	*Versicolores*

	CCMFBH-889<br>CCMFBH-899<br>CCMFBH-916<br>CCMFBH-960<br>CCMFBH-973<br>CCMFBH-974<br>CCMFBH-987<br>CCMFBH-990	MT410090<br>MT410091<br>MT410092<br>MT410093<br>MT410094<br>MT410095<br>MT410096<br>MT410097		
**XVII**	*KJ775092.1-*A. templicola*<br>KJ775087.1-*A. templicola*<br>CCMFBH-967	 <br>MT410171	*A. templicola*	*Flavipedes*
**XVIII**	*KJ775085.1-*A. micronesiensis*<br>CCMFBH-920<br>CCMFBH-965<br>CCMFBH-969	 MT410158<br>MT410170<br>MT410172	*A. micronesiensis*	*Flavipedes*
**XIX**	*MG991317.1-*A. tamarii*<br>*MH279885.1-*A. tamarii*<br>CCMFBH-850<br>CCMFBH-853<br>CCMFBH-854<br>CCMFBH-895<br>CCMFBH-902<br>CCMFBH-911<br>CCMFBH-924<br>CCMFBH-933<br>CCMFBH-976<br>CCMFBH-985<br>CCMFBH-1000	 <br>MT410126<br>MT410127<br>MT410128<br>MT410148<br>MT410150<br>MT410152<br>MT410159<br>MT410162<br>MT410174<br>MT410176<br>MT410182	*A. tamarii*	*Flavi*
**XX**	*AF255067.1-*A. nomius*<br>*KP418578.1 *A. nomius*<br>CCMFBH-866	 <br>MT410133	*A. nomius*	*Flavi*
**XXI**	*F669489.1 *A. oryzae*<br>CCMFBH-888	 MT410145	*A. oryzae*	*Flavi*
**XXII**	*MN148880.1 *A. flavus*<br>*KY272750.1 *A. flavus*<br>CCMFBH-837<br>CCMFBH-847<br>CCMFBH-856<br>CCMFBH-858<br>CCMFBH-870<br>CCMFBH-891	 <br>MT347711<br>MT347712<br>MT347713<br>MT347714<br>MT347715<br>MT347716	*A. flavus*	*Flavi*

	CCMFBH-892<br>CCMFBH-896<br>CCMFBH-918<br>CCMFBH-923<br>CCMFBH-925<br>CCMFBH-929<br>CCMFBH-938<br>CCMFBH-948<br>CCMFBH-956<br>CCMFBH-968<br>CCMFBH-970<br>CCMFBH-984<br>CCMFBH-986<br>CCMFBH-992	MT347717<br>MT347718<br>MT347719<br>MT347720<br>MT410160<br>MT347721<br>MT347722<br>MT347723<br>MT347724<br>MT347725<br>MT347726<br>MT347727<br>MT347728<br>MT347729		
**XXIII**	*MG991380.1-*A. fumigatus*<br>*MG991403.1-*A. fumigatus*<br>CCMFBH-876<br>CCMFBH-878<br>CCMFBH-921<br>CCMFBH-953<br>CCMFBH-958<br>CCMFBH-961<br>CCMFBH-962<br>CCMFBH-963<br>CCMFBH-977<br>CCMFBH-978	 <br>MT347701<br>MT347702<br>MT347703<br>MT347704<br>MT347705<br>MT347706<br>MT347707<br>MT347708<br>MT347709<br>MT347710	*A. fumigatus*	*Fumigati*
**XXIV**	*EU076336.1-*A. giganteus*<br>*EU076337.1-*A. giganteus*<br>*EU076338.1-*A. giganteus*<br>CCMFBH-859<br>CCMFBH-860	 <br><br>MT410129<br>MT410130	*A. giganteus*	*Clavati*
**XXV**	* KX455765.1-*A. clavatus*<br>* KF669483.1 -*A. clavatus*<br>CCMFBH-841<br>CCMFBH-842<br>CCMFBH-843<br>CCMFBH-844<br>CCMFBH-863<br>CCMFBH-941<br>CCMFBH-945<br>CCMFBH-1001	 <br>MT410121<br>MT410122<br>MT410123<br>MT410124<br>MT410132<br>MT410165<br>MT410166<br>MT410183	*A. clavatus*	*Clavati*
**XXVI**	*HQ632671.1 *A. aculeatus*		*A. aculeatus*	***N**igri*

	CCMFBH-993	MT410178		
**XXVII**	*FJ491685.1-*A. violaceofuscus*<br>*FJ491686.1-*A. violaceofuscus*<br>CCMFBH-893	 <br>MT410147	*A. violaceofuscus*	*Nigri*

* Reference sequences used for maximum likelihood analysis
