# Supplementary figures and images for: Phylogenetic Identification, Diversity, and Richness of Aspergillus from Homes in Havana, Cuba
Source: Microorganisms. 2021 Jan 6;9(1):115. doi: 10.3390/microorganisms9010115 (PMC7825327; doi:10.3390/microorganisms9010115)

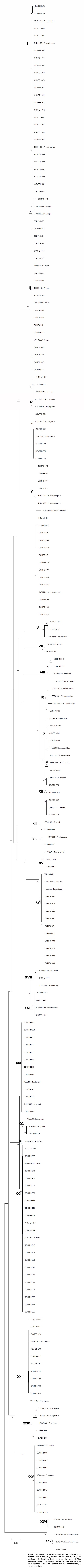

Supplement: Supplementary file 1 [file microorganisms-09-00115-s001.zip › microorganisms-1059983-sl-revised/Supplementary Figure S1 S¿ónchez Espinosa et al..jpg]
